# Near-Infrared Phosphorescence Emission of Binuclear Mn(II) Based Metal-Organic Framework for Efficient Photoelectric Conversion

**DOI:** 10.3389/fchem.2020.593948

**Published:** 2020-11-11

**Authors:** Mei-Li Zhang, Zhi-Min Zhai, Xiao-Gang Yang, Ya-Dan Huang, Yan-Jin Zheng, Lu-Fang Ma

**Affiliations:** ^1^Laboratory of New Energy and New Function Materials, Department of Chemistry and Chemical Engineering, Yan'an University, Yan'an, China; ^2^Henan Province Function-Oriented Porous Materials Key Laboratory, College of Chemistry and Chemical Engineering, Luoyang Normal University, Luoyang, China; ^3^College of Chemistry and Chemical Engineering, Henan Polytechnic University, Jiaozuo, China

**Keywords:** metal-organic framework, room temperature phosphorescence, photoelectron performance, host-guest, π-stacking

## Abstract

The development of metal-organic framework (MOF) based room-temperature phosphorescence (RTP) materials has raised extensive concern owing to their widespread applications in the field of anti-counterfeiting, photovoltaics, photocatalytic reactions, and bio-imaging. Herein, one new binuclear Mn(II) based 3D MOF [Mn_2_(L)(BMIB)·(H_2_O)] (**1**) (H_5_L = 3,5-bis(3,5-dicarboxylphenxoy) benzoic acid, BMIB = tran-4-bis(2-methylimidazolyl)butylene) has been synthesized by a facile hydrothermal process. In **1**, the protonated BMIB cations show infinite π-stacking arrangement, residing in the channels of the 3D network extended by L ligand and binuclear Mn(II) units. The orderly and uniform host-guest system at molecular level emits intense white light fluorescence and long-lived near infrared phosphorescence under ambient conditions. These photophysical processes were well-studied by density functional theory (DFT) calculations. Photoelectron measurements reveal high photoelectron response behavior and incident photon-to-current efficiency (IPCE).

## Introduction

Room-temperature phosphorescence (RTP) materials have been widely used in a variety of optoelectronic fields such as photovoltaics, photocatalytic reactions and molecular sensing, owing to its long-lived excited states, which can increase the production of free charges (Mukherjee and Thilagar, 2015; Yang and Yan, 2016b; Yang et al., 2018, 2019a,b, 2020a; Gu et al., 2019; Yuan et al., 2019; Zhao et al., 2019; Zhou and Yan, 2019). Generally, key factors to improve the phosphorescence performance can be concluded as follows: (a) promote the intersystem crossing by increasing spin–orbit coupling through the introduction of hetero atoms, heavy atoms; (b) inhibit the non-radiative decay of triplet exciton by the formation of crystallization and the rigid matrix (Bolton et al., 2011; Gong et al., 2015; Kabe et al., 2016; Baryshnikov et al., 2017; Kabe and Adachi, 2017; Li and Li, 2017; Liu et al., 2017; Xie et al., 2017; Yang et al., 2018; Hirata, 2019; Ma and Tian, 2019; Mao et al., 2019). In this sense, metal-organic frameworks (MOFs) can be considered as a outstanding platform to achieve efficient RTP owing to their functionally adjustable structure and various topology matrices (Wang et al., 2016, 2019; Tan et al., 2018; Qin et al., 2019; Wu et al., 2019; Chen et al., 2020; Yang et al., 2020b). As a kind of crystalline material, the spatial confinement effect of MOFs can maximally increase the rigidity of molecular conformations, suppressing the molecular motions/vibrations for the chromophore linkers or guests. For example, Adachi's group reported persistent RTP emission up 22.4 s to can be obtained by encapsulating coronene into cages of ZIF-8 (Mieno et al., 2016). The interposition and volatilization of guest solvent molecules can also result in the reversible transformation of phosphorescence emission (Yang and Yan, 2016a). Considering the variety of MOFs, it still remains a challenge to study relationships between the RTP performance and structures of such materials.

**Scheme 1:**
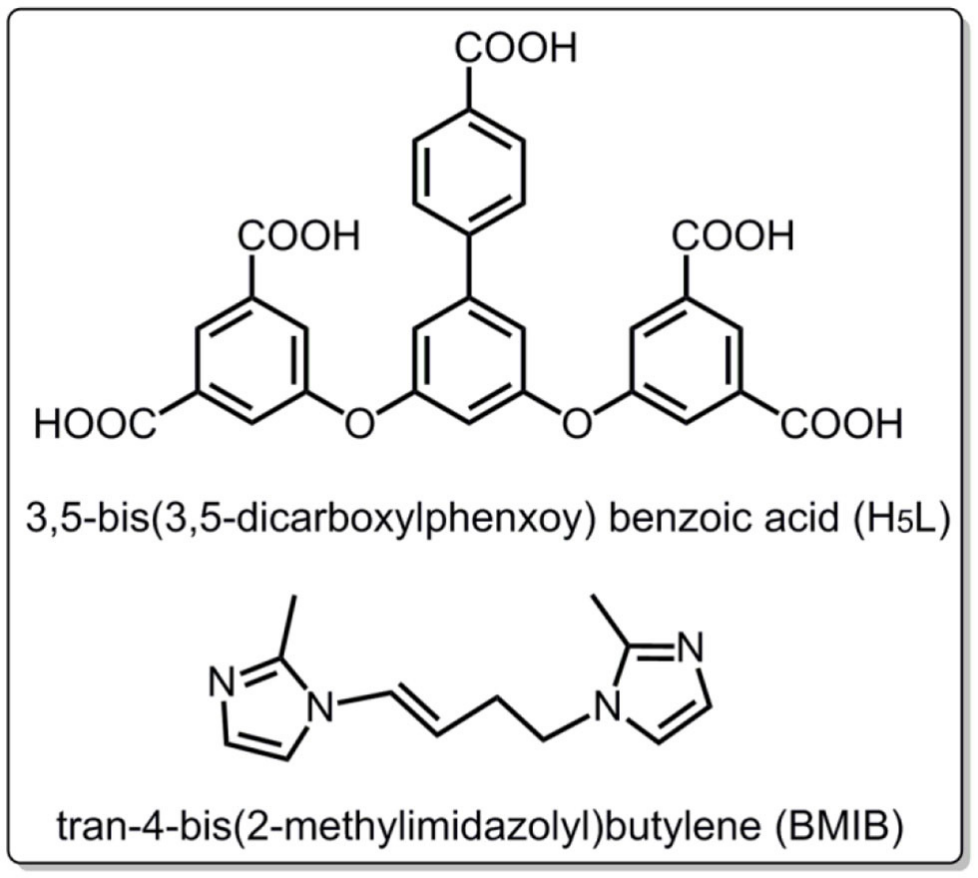
Chemical structures of H_5_L and BMIB molecules in this work.

In this paper, we reported one new binuclear Mn(II) based MOF, [Mn_2_(L)(BMIB)·(H_2_O)] (**1**), by selection of polycarboxylate ligand 3,5-bis(3,5-dicarboxylphenxoy) benzoic acid (H_5_L) and an N-heterocyclic ligand tran-4-bis(2-methylimidazolyl)butylene (BMIB) as shown in Scheme 1. The title MOF exhibits a 3D network with the π-stacking BMIB cations residing in the channels. Steady/transient state measurements indicate that the orderly and uniform arrangement of Mn-L 3D host and BMIB guests exhibits intense white light fluorescence and long lifetime of near infrared phosphorescence emission under ambient conditions. The density functional theory (DFT) calculations and photoelectron behavior have also been studied.

## Experimental

### Materials and General Methods

All reagents were of analytical grade and obtained from commercial sources without further purification. PXRD patterns were collected on a Bruker D8-ADVANCE X-ray diffractometer with Cu *K*α radiation (λ = 1.5418 Å) with a step of 0.02° (2θ). The C, H, N analyses were carried out using a Perkin–Elmer Elementarvario elemental analysis instrument. Thermogravimetric analysis (TGA) experiments were carried out using SII EXSTAR6000 TG/DTA6300 thermal analyzer from room temperature to 800°C under a nitrogen atmosphere at a heating rate of 10°C min^−1^. The IR spectra was recorded in the range of 4,000–400 cm^−1^ on a Nicolet 6700 (Thermo) FT-IR spectrometer with KBr pellets. Room temperature photoluminescence spectra and decay curves were measured by Edinburgh FLS1000 fluorescence spectrometer with a xenon arc lamp (Xe900), and nano/microsecond flashlamp. UV-vis absorption spectra was measured by Shimadzu UV-3600 plus UV-vis-NIR spectrophotometer.

Electrocatalytic measurements were conducted by CHI 660E electrochemical workstation in 0.5 M Na_2_SO_4_ solution at room temperature. Monochromatic light was generated using the Omni-λ 150 monochromator, and the output power was measured using a photodiode detector. The incident photon-to-current efficiency (IPCE) at each wavelength was measured by MPI-EO PEC analysis system (Xi'an Remex Analysis Instrument Co., Ltd., Xi'an, China) with external potential at −0.5 V vs. Ag-AgCl. The monochromatic light was generated by a 300 W Xe arc lamp assembled with a Omni-λ150 monochromator. IPCE = (1,240 *I*)/(λ *P*_light_), where *I* is the photocurrent density (mA cm^−2^), λ is the incident light wavelength (nm), and *P*_light_ is the power density of monochromatic light at each wavelength (mW cm^−2^).

### Syntheses of [Mn_2_(L)(BMIB)·(H_2_O)] (1)

A mixture of MnSO_4_·H_2_O (0.5 mmol, 84.5 mg), 3,5-bis(3,5-dicarboxylphenxoy) benzoic acid (0.25 mmol, 119 mg), tran-4-bis(2-methylimidazolyl)butylene (0.25 mmol, 54.2 mg) and 8 mL of H_2_O was stirred for 10 min. The mixture was then transferred and sealed into a Teflon reactor (23 ml), and heated at 150°C for 48 h, and then cooled to room temperature naturally. Colorless block crystals of **1** were obtained. Yield: 60% (based on Mn). Anal. Calc. (%) for C_35_H_28_Mn_2_N_4_O_13_: C 51.11, H 3.43, N 6.84; found (%): C 50.97, H 3.25, N 6.69. IR (KBr pellet, cm^−1^): 3,422 m, 1,960 w, 1,608 m, 1,545 m, 1,446 m, 1,388 s, 1,068 m, 966 w, 771 w.

### X-Ray Crystal Structure

Crystal Structure was tested on Oxford Diffraction SuperNova area-detector diffractometer with the program of CrysAlisPro and solved by SHELXS-2014 and SHELXL-2014 software (Sheldrick, 2008, 2015). The crystallographic data for **1** is listed in Table 1. CCDC No. 2011677 contains the supplementary crystallographic data for **1**.

**Table 1:** Crystallographic data for **1**.

**Sample**	**1**
Chemical formula	C_35_H_28_Mn_2_N_4_O_13_
Formula weight	822.5
Crystal system	triclinic
Space group	$P_{\bar{1}}$
*a* (Å)	9.7440 (4)
*b* (Å)	10.8967 (4)
*c* (Å)	17.8019 (6)
α (°)	92.251 (3)
β (°)	97.727 (3)
γ (°)	114.333 (4)
*V* (Å^3^)	1697.20 (12)
*Z*	2
*D* (g cm^−3^)	1.6093
μ (mm^−1^)	0.820
*R*_int_	0.0223
Goof	1.050
$R_{1}^{\text{a}}$ (*I>2σ* (*I*))	0.0499
*wR_2_*^b^ (*I>2σ*(*I*))	0.1222

a *R_1_ = *Σ*(||F_o_| **–** |F_c_||)/*Σ*|F_o_|*;

b *wR_2_ = [*Σ*w(|F_o_|^2^**–** |F_c_|^2^)^2^/*Σ*w(${\text{F}}_{\text{o}}^{2}$)^2^]^1/2^*.

## Results and Discussion

### Crystal Structure Description

**Figure 1:**
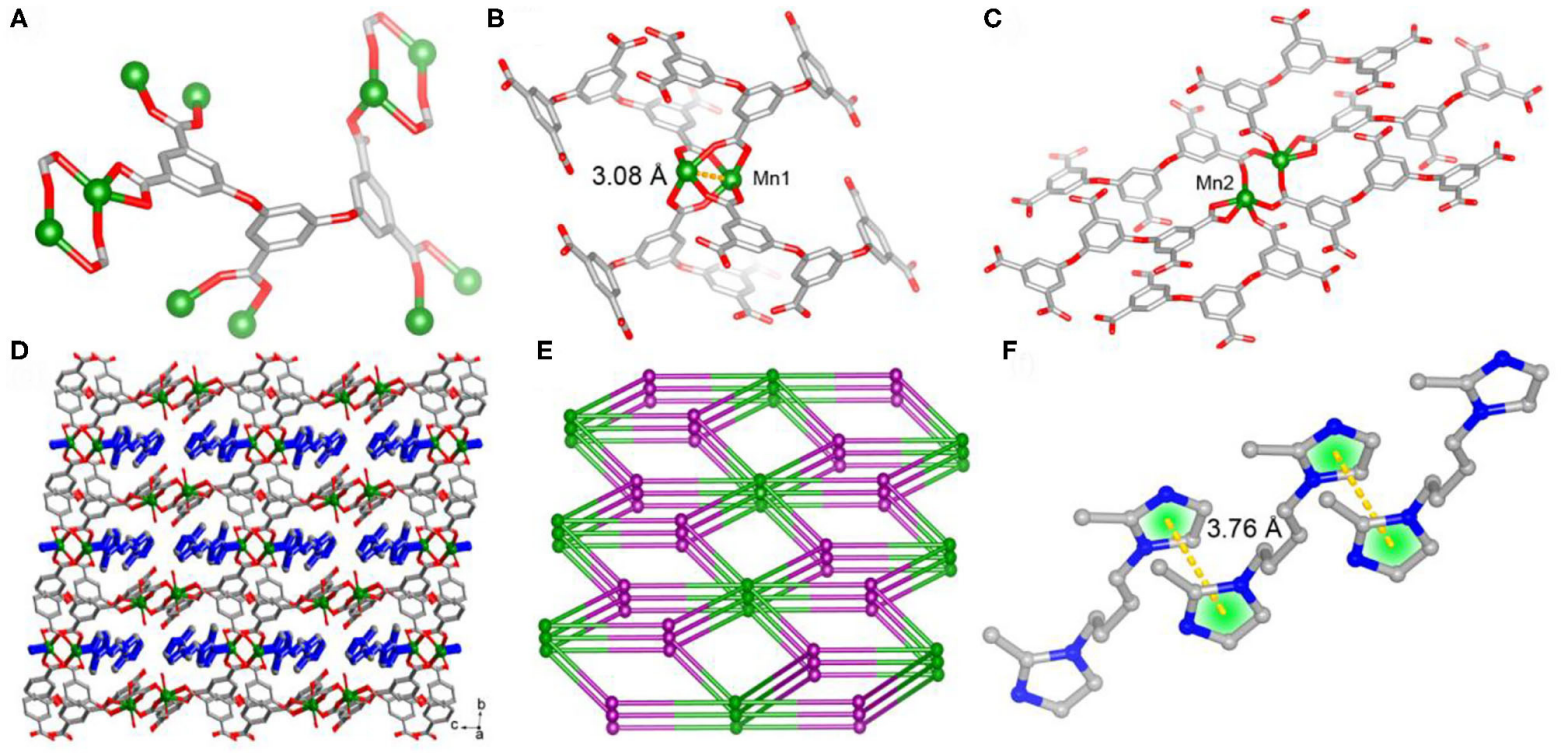
The coordination modes of L ligand **(A)** and binuclear Mn(II) clusters **(B)** and **(C)** in **1**. **(D)** View of the 3D network of **1** with protonated BMIB ligands fixed in the channels through coordination bonds. **(E)** Schematic illustration of 3D network of **1**. **(F)** π-stacking between BMIB ligands.

Single-crystal X-ray diffraction analysis reveals that **1** crystallizes in triclinic $P_{\bar{1}}$ space group, the asymmetric unit of which consists of two independent Mn(II) atoms, one L anion and one protonated BMIB. In **1**, the completely deprotonated ligand polycarboxylate ligand 3,5-bis(3,5-dicarboxylphenxoy) benzoate displays twisty spatial configuration with the torsion angles between two arm benzene rings and the central one of 82.9 and 78.5°, respectively. The five carboxylate groups show monodentate, bidentate and chelate coordination modes, connecting eight Mn(II) atoms (Figure 1A). On the other hand, there are two kind of binuclear Mn(II) units, four bidentate carboxylates link the metal dimmer to form a paddle-wheel cluster with half-coordinated BMIB ligands loading on the axial position and short Mn···Mn distance of 3.08 Å. While the other binuclear Mn(II) unit shows longer Mn···Mn distance of 3.69 Å. The paddle-wheel cluster is surrouded by four L liagands (Figure 1B), the later binuclear Mn(II) unit is coordinated by six L ligands (Figure 1C). Based on above coordination fashion, these Mn(II) clusters are extended by L ligands giving rise to a 3D network. It is worth noting that the BMIB ligands possess one protonated and one monodentate coordinated imidazole ring, which act as guests and charge compensating units, and are fixed in the channels of the 3D network through coordination and electrostatic interactions (Figure 1D). Topological analysis show that the title MOF can be simplified as a (4,5,6)-connected net (Figure 1E). It is found that the BMIB ligands are arranged in a 1D continuous π···π stacking between imidazole rings with the centroid to centroid distance of 3.76 Å (Figure 1F). The above structure feature suggests that the confined π-conjugate system would provide an efficient electron delivery pathway along the channels.

### PXRD and Thermal Gravimetric Analysis

**Figure 2:**
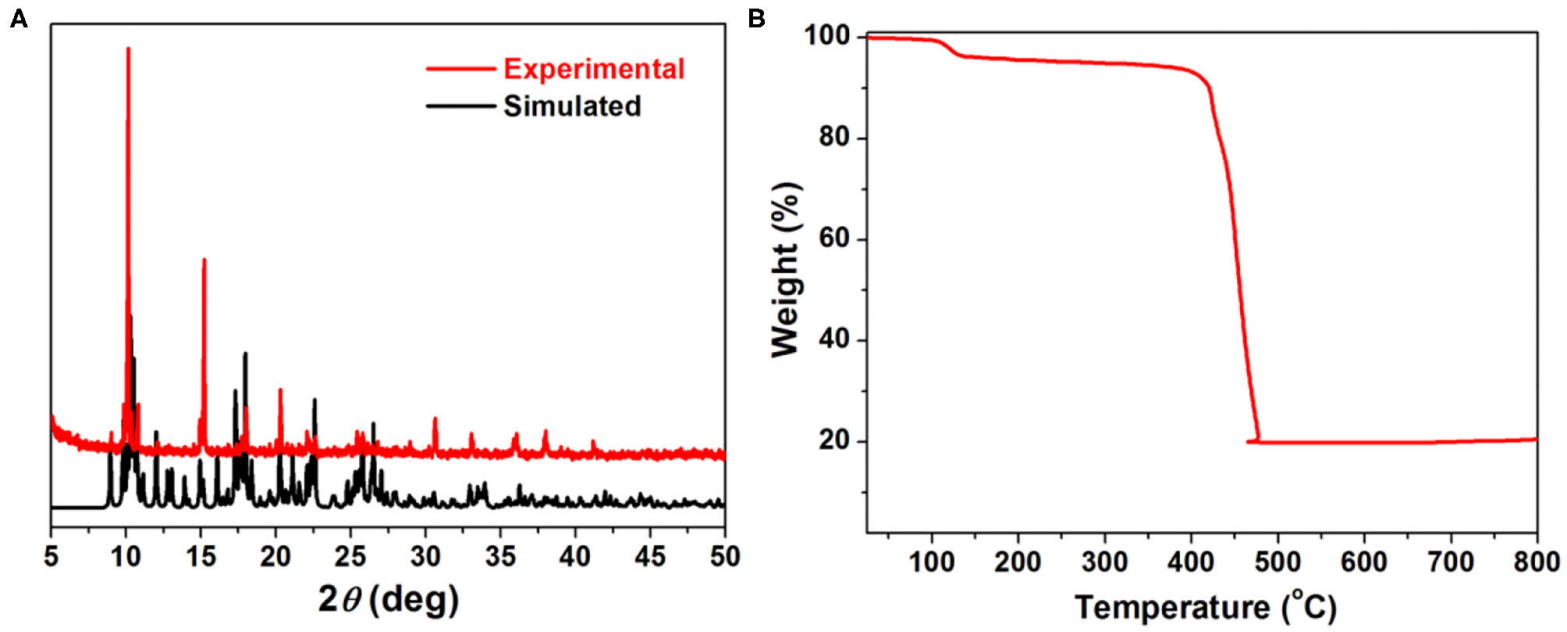
**(A)** PXRD patterns of simulated (black) and as synthesized **1** (red). **(B)** Thermo gravimetric analysis curve of **1**.

The phase purity of title MOF was characterized by powder X-ray diffraction analyses (PXRD) as shown in Figure 2A. The position of the main diffraction peaks of the experiment match well with the simulated one, indicating the high purity and crystalline of the as-synthesis samples. The thermo gravimetric analysis (TGA, Figure 2B) curve shows the first weight loss of about 2.18% in the range of 100–130°C, which can be assigned to the loss of coordinated water molecules (calculated: 2.78%). By additional heating, the framework of **1** can be stable up to about 400°C, indicating a high thermal stability of such MOF.

### Photoluminescence Properties

**Figure 3:**
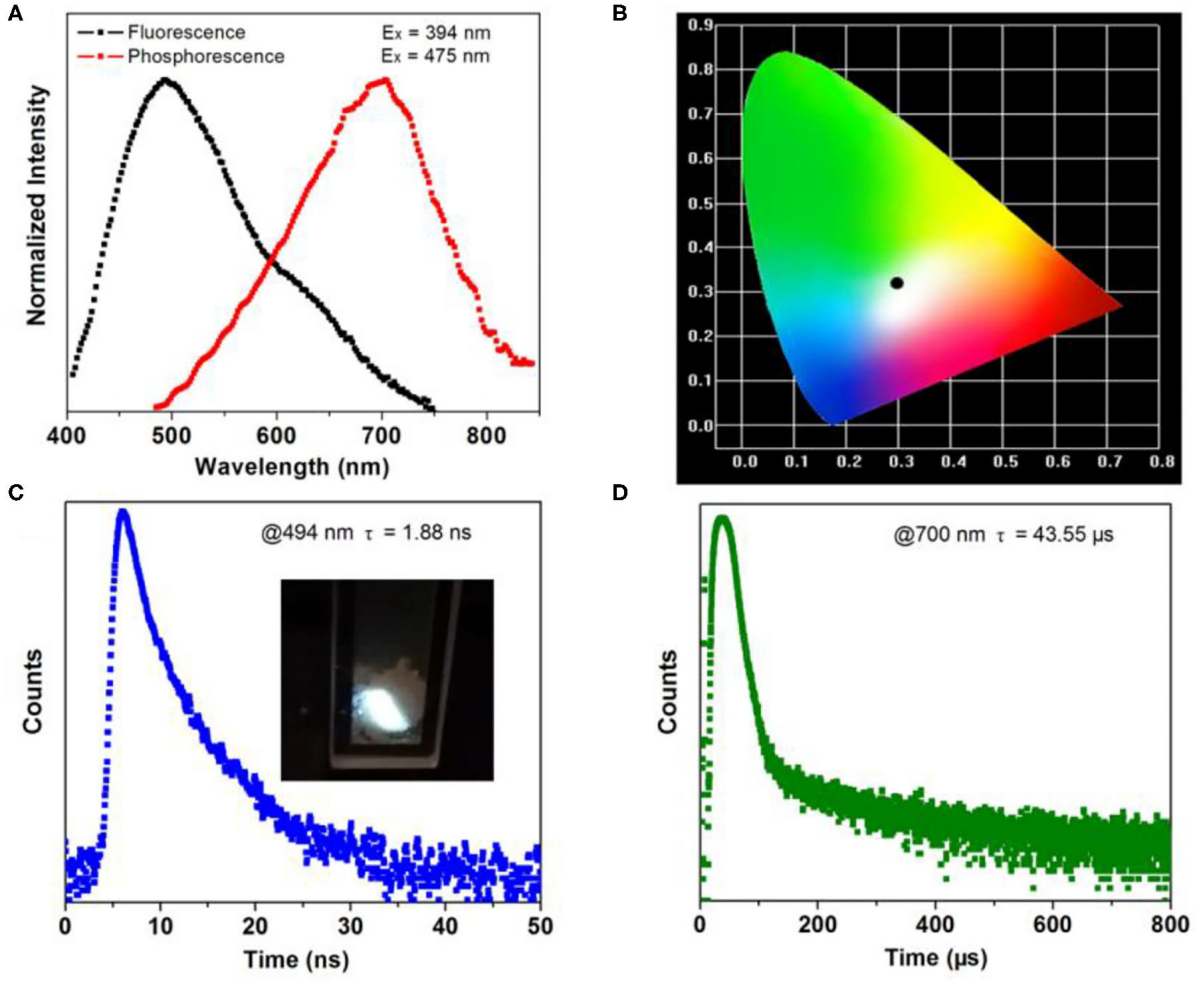
**(A)** Normalized fluorescence/phosphorescence spectra of **1**. **(B)** CIE-1931 chromaticity diagram of **1**. Fluorescence **(C)** and phosphorescence **(D)** decay curves of **1** measured at room temperature. Insets show the crystal sample of **1** under UV (365 nm) light.

The photoluminescence measurement of the crystalline material was conducted at room temperature. The emission of **1** peaks at 494 nm spanning a broad range from 400 to 750 nm when excited by 394 nm (Figure 3A). The chromaticity coordinate of (0.301, 0.315) indicates white emission of **1** (Figure 3B). From the insert in Figure 3C, the crystal sample of **1** emits intense white fluorescence under 365 nm UV lamp irradiation. Under the excitation of 475 nm microsecond flashlamp, long-wavelength of phosphorescence spectra peak at 700 nm can be detected. This results in a large Stocks shift of 48,543 cm^−1^ in comparison with the fluorescence emission. Further photoluminescence decay curves show a short lifetime of 1.88 ns for fluorescence emission (measured at 494 nm) and a long one of 43.55 μs for phosphorescence emission (measured at 700 nm). As a result, **1** features both energy (emission wavelength) and time (emission lifetime) scale diversity stimulated by the continuous and pulsing light source.

### Density Functional Theory (DFT) Calculations

**Figure 4:**
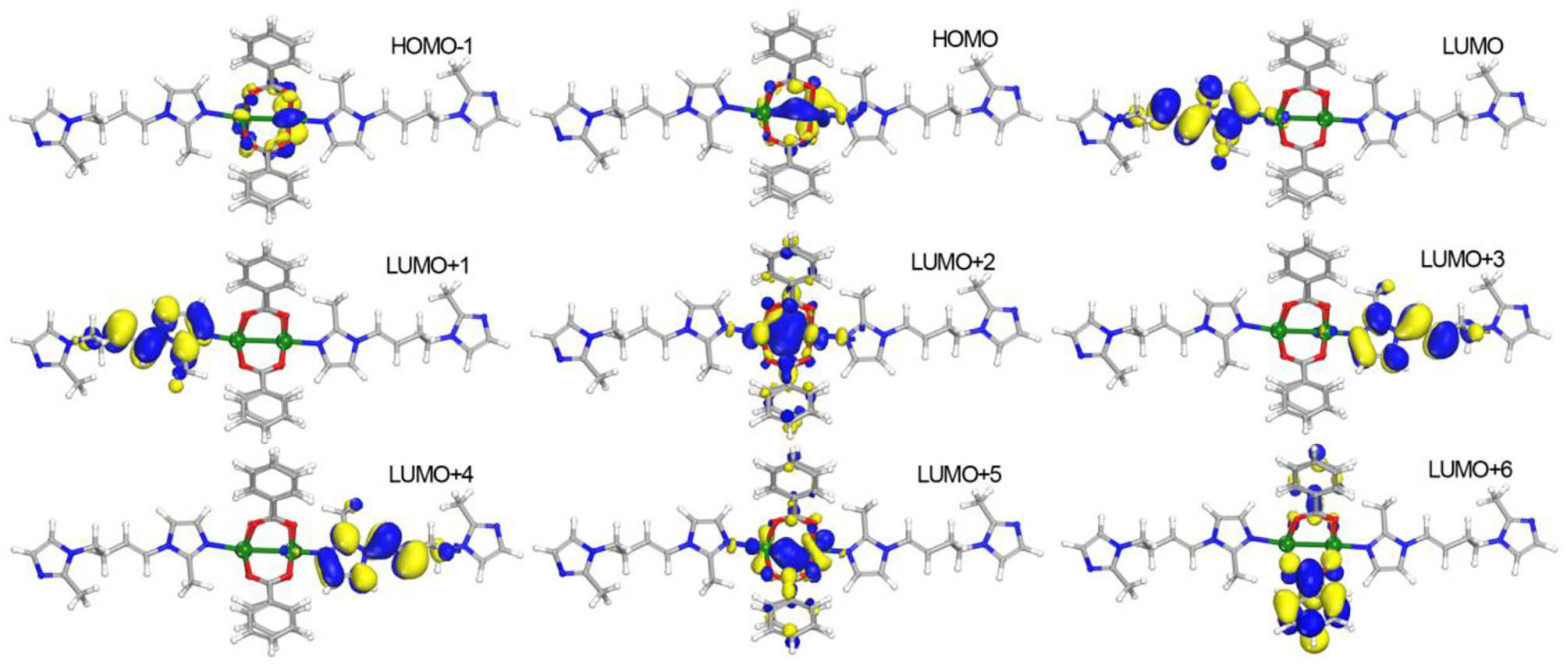
View of the distributions of highest occupied molecular orbitals (HOMOs) and lowest unoccupied molecular orbitals (LUMOs) for **1**.

To further understand the photophysical process of **1**, density functional theory (DFT) calculations were performed by Dmol^3^ module in Material Studio software package (Delley, 2000). The structure mode was set from the crystallographic information file (cif) of **1** by removing the symmetry and leaving the binuclear Mn(II) unit. The calculated results (Figure 4) show that the highest occupied molecular orbitals (HOMOs) exclusively distribute on Mn(II) atoms. The lowest unoccupied molecular orbitals (LUMOs) including LUMO, LUMO+1, LUMO+3, and LUMO+4 mainly appear on BMIB ligands. It is worth noting that the electronic isodensity surfaces are located between two Mn(II) atoms in LUMO+2 and LUMO+5, indicating the delocalization of *d* electron in the Mn(II) cluster. The LUMO+6 mainly disperse in the benzoate. Based on the above analysis, it can be concluded that the photophysical process of **1** contains the mixture of metal-centered luminescence and metal to ligand charge transfer (MLCT).

### Photoelectron Performance

**Figure 5:**
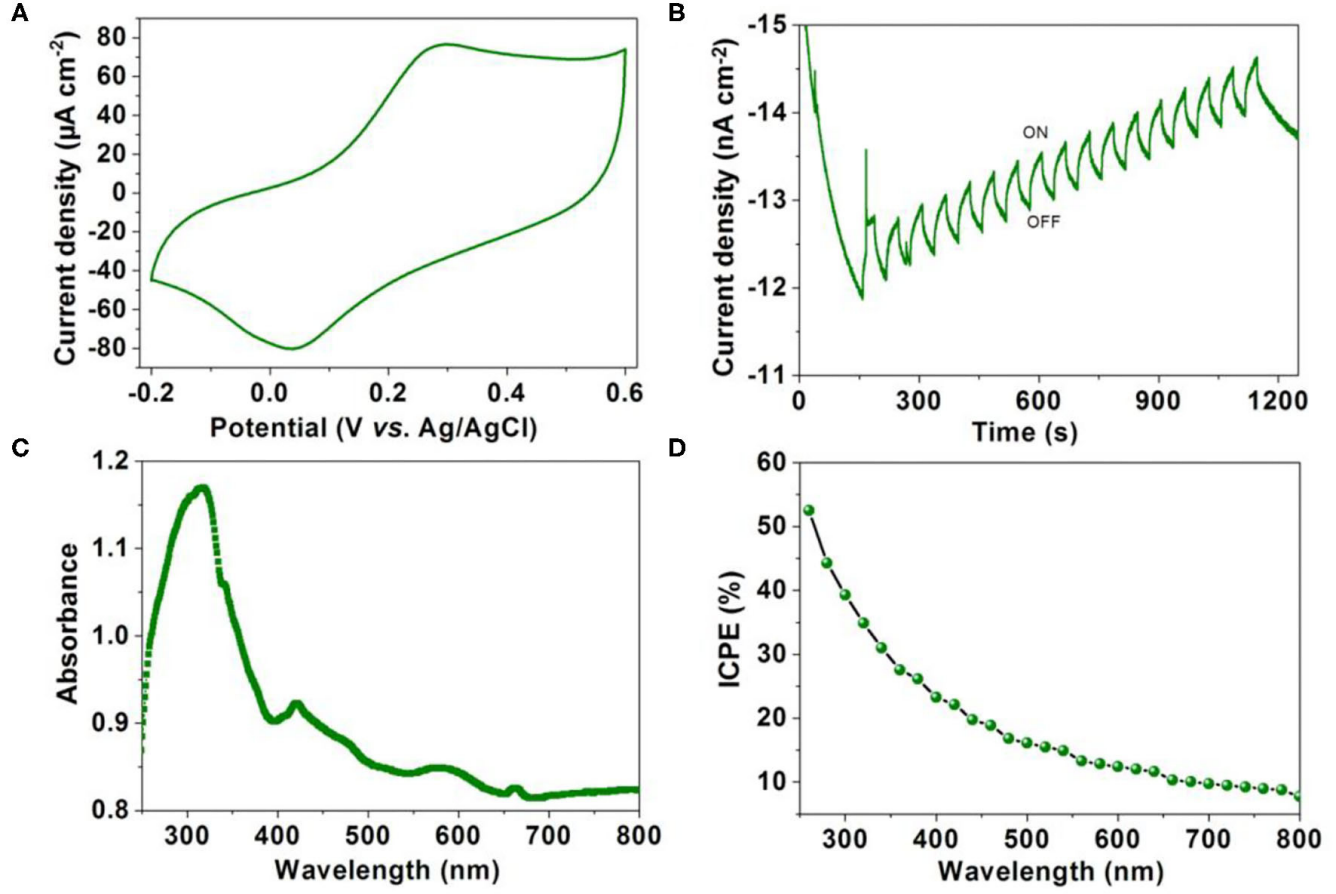
**(A)** Cyclic voltammograms cuves of **1** modified ITO electrode measured in 0.5 M Na_2_SO_4_ aqueous solution. **(B)** Transient current density–time curve of **1** at a bias potential of −0.5 V with the periodic on-off cycles of illumination. **(C)** The UV-visible absorption spectrum of **1**. **(D)** Action spectrum for IPCE vs. wavelength of **1**.

It has been demonstrated that long lifetime of the triplet state exciton enables the slow recombination rate of electrons and holes. Photoelectron performance was measured by a three-electrode system in Na_2_SO_4_ solution (0.5 M) with the MOF modified indium tin oxide (ITO) glass as the working electrode. The cyclic voltammogram curve shows that **1** has good electrochemical activity (Figure 5A). As shown in Figure 5B, the current generated by **1** modified indium tin oxide (ITO) electrode shows a gradually increasing trend at a bias potential of −0.5 V with the periodic on-off cycles of illumination, suggesting efficient photoelectron response performance. The UV-visible absorption spectrum (Figure 5C) reveals that the intense absorption band around 316 nm can be assigned to intra-ligand (π → π^*^) transitions, whereas the less intense peak at 419 nm is attributed to the metal-to-ligand charge-transfer (MLCT). Further incident photon-to-current efficiency (IPCE) vs. the wavelength curve shows a high IPCE of 53% at a bias potential of −0.5 V (Figure 5D). The above results indicate **1** can be used as an efficient photoelectric conversion, CO_2_ reduction and H_2_O oxidation material (Fu et al., 2018; Chen et al., 2020; Lu et al., 2020; Xu et al., 2020). It also provides a new idea for the application of MOF-based materials.

## Conclusions

In summary, one new binuclear Mn(II) based 3D MOF host-guest material can be facilely synthesized by the assemble of flexible polycarboxylate ligand 3,5-bis(3,5-dicarboxylphenxoy) benzoic acid (H_5_L) and N-heterocyclic ligand tran-4-bis(2-methylimidazolyl)butylene (BMIB). The orderly arrangement BMIB π-conjugated chains are confined in the MOF channels through coordination and electrostatic interactions, providing a structure model of molecular level heterojuction for high performance of white light fluorescence and long-lived near infrared phosphorescence emission as well as photoelectron conversion. Therefore, this work not only provides a facial process to obtain near-infrared phosphorescence emission material, but also proposes new opportunities to introduce phosphorescence MOF materials for potential optoelectronic applications.

## Data Availability Statement

The original contributions presented in the study are included in the article/supplementary material, further inquiries can be directed to the corresponding author/s.

## Author Contributions

X-GY and L-FM conceived the idea and designed research. M-LZ, Z-MZ, Y-DH, and Y-JZ synthesized and characterized materials. All authors analyzed data and wrote the paper. All authors contributed to the article and approved the submitted version.

## Conflict of Interest

The authors declare that the research was conducted in the absence of any commercial or financial relationships that could be construed as a potential conflict of interest.
